# UTILE-Gen: Automated Image Analysis in Nanoscience Using Synthetic Dataset Generator and Deep Learning

**DOI:** 10.1021/acsnanoscienceau.3c00020

**Published:** 2023-08-02

**Authors:** André Colliard-Granero, Jenia Jitsev, Michael H. Eikerling, Kourosh Malek, Mohammad J. Eslamibidgoli

**Affiliations:** † Theory and Computation of Energy Materials (IEK-13), Institute of Energy and Climate Research, Forschungszentrum Jülich GmbH, 52425 Jülich, Germany; ‡ Centre for Advanced Simulation and Analytics (CASA), Simulation and Data Science Lab for Energy Materials (SDL-EM), Forschungszentrum Jülich GmbH, 52425 Jülich, Germany; § Chair of Theory and Computation of Energy Materials, Faculty of Georesources and Materials Engineering, RWTH Aachen University, 52062 Aachen, Germany; ∥ Jülich Supercomputing Center, Forschungszentrum Jülich, 52425 Jülich, Germany

**Keywords:** nanoscience, deep learning, convolutional neural networks, synthetic data, domain randomization, particle analysis

## Abstract

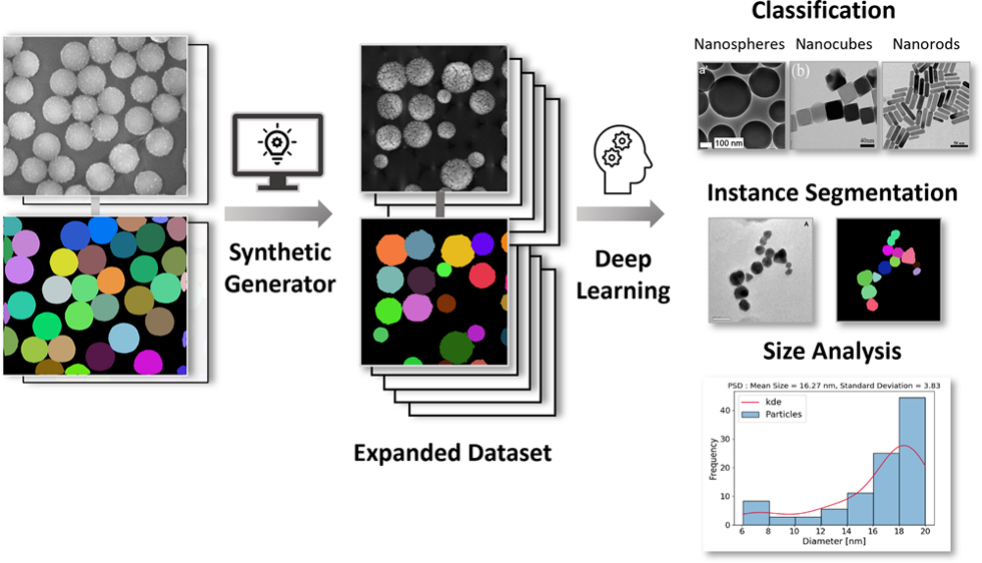

This work presents the development and implementation of a deep learning-based workflow for autonomous image analysis in nanoscience. A versatile, agnostic, and configurable tool was developed to generate instance-segmented imaging datasets of nanoparticles. The synthetic generator tool employs domain randomization to expand the image/mask pairs dataset for training supervised deep learning models. The approach eliminates tedious manual annotation and allows training of high-performance models for microscopy image analysis based on convolutional neural networks. We demonstrate how the expanded training set can significantly improve the performance of the classification and instance segmentation models for a variety of nanoparticle shapes, ranging from spherical-, cubic-, to rod-shaped nanoparticles. Finally, the trained models were deployed in a cloud-based analytics platform for the autonomous particle analysis of microscopy images.

## Introduction

The development of deep learning (DL) algorithms to automate image processing and image analysis is one of the most rapidly growing fields in computer vision (CV).^1^ Convolutional neural networks (ConvNets) are among the state-of-the-art methodologies that outperform classical algorithms in a wide variety of image recognition tasks, including verification/identification, classification, object detection, segmentation, image reconstruction, denoising, colorization, or style transfer.^2,3^ ConvNets have also been applied to solve various problems in materials research,^4^ such as high-throughput classification of microscopy images of catalyst materials and their particle size distribution analysis,^5^ detecting defects in nanofibrous materials,^6^ and high-resolution synchrotron tomography by denoising reconstructed images of internal material structures.^7^

Instead of performing several handcrafted preprocessing steps, DL models—trained on representative datasets—can learn to extract complex features directly from the raw micrographs, enabling automation in the analysis. Furthermore, well-defined performance metrics validate the reliability of such models, providing robust quantitative standards. However, one of the major challenges for supervised model training tasks is the availability of labeled data. This issue is exacerbated in highly specialized domains, such as nanoscience, where the lack of annotated datasets hinders the building of autonomous analytics tools.^8^

Electron microscopy (EM) is one of the most utilized characterization techniques in nanoscience.^9^ Based on the image analysis, nanoparticle (NP) images can be characterized by a variety of parameters, including shape, size, or spatial distribution. After collecting samples and imaging data, researchers often perform particle measurements manually using image analysis software such as Fiji.^10^ This approach, however, is tedious and inefficient for high-throughput or real-time analysis due to its slow, laborious, and highly specialized manual procedure.

Semiautomated methods based on classical computer vision algorithms were integrated into the image analysis software, but they are often applicable for simple cases where particles are well segregated or monodispersed. In these methods, conventional procedures like Otsu’s binarization and Canny edge detection were adopted for detecting the particle boundaries.^11^ Other techniques have also been developed based on the template matching strategy, which assigns a score to each probable particle location in the image.^12,13^ Such methods, however, are not often applicable to analyze complex cases with different particle shapes, distributions, or heterogeneous image backgrounds. Furthermore, two-dimensional (2D) mapping complicates semiautomated thresholding techniques for segmenting overlapping and crowded particle systems.

The application of DL algorithms for the analysis of EM data has been thoroughly reviewed in a recent article.^14^ For example, the U-Net architecture^15^ with StarDist formulation for loss function^16^ were trained to automate the particle size distribution analysis of electrocatalyst materials, where various shape, texture, and patterns are generated between the overlapping catalyst NPs and the support material.^17^ DL models were also used for real-time segmentation of NPs in liquid phase EM movies to statistically examine the diffusion, reactivity, and assembly kinetics of cube-, prism-, and rod-shaped colloidal NPs.^18^ Another example is the ImageDataExtractor software which uses Bayesian DL to segment and quantify NPs of different morphologies.^19^

**Figure 1:**
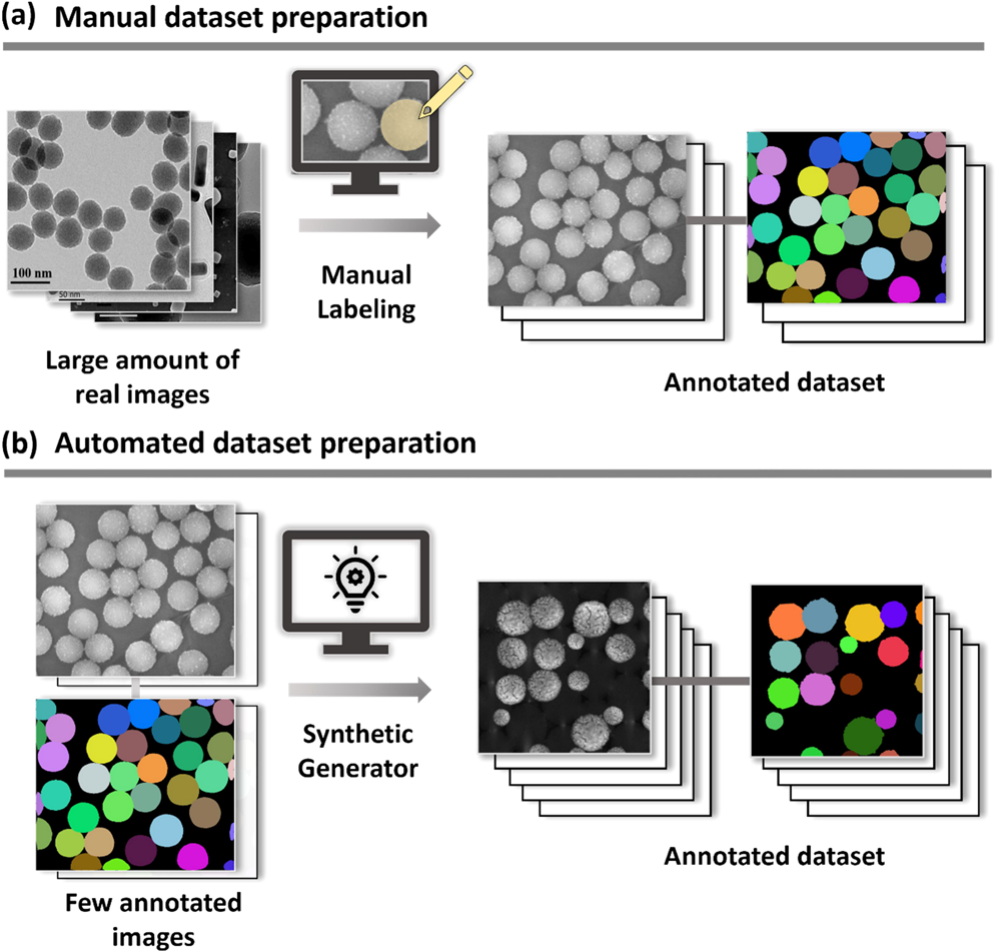
Dataset preparation for supervised model training tasks. Time-consuming, manually performed single-particle annotation in (a) is compared with automated synthetic dataset generation with expanded datasets and instance segmentation masks in (b).^27^

Supervised DL algorithms for image classification and segmentation require a large amount of annotated and high-quality data for training. While the manual annotation of the regions of interest (ROIs) in the image is time-consuming, various approaches were reported for the synthetic dataset generation (Figure 1). For DL-based image synthesis, generative adversarial networks (GANs) are prevalent.^20^ Notably, Zhang et al. developed the DatasetGAN,^21^ which generates semantic segmentation masks in addition to synthetic images. The training of GANs is, however, computationally expensive, and they also require large datasets. Other reported techniques are based on data augmentation methods like domain randomization (DR). Applying DR on 2D images is based on cropping of ROI from the original image and randomly pasting them on a similar background after applying geometrical transformations like flipping, rotating, or resizing. Simultaneously, segmentation masks can be created. These simple procedures can provide effective datasets for training DL models and are also adaptable to various systems.

Even though the synthetic images using DR look less realistic than those generated with GANs, there have been several reports of the effective use of such methods for training the DL models. For example, the “Cut, Paste, and Learn” method was proposed by Dwibedi et al.^22^ for object detection. Toda et al.^23^ applied DR for segmented dataset generation of seeds; here, a pool of different seeds was created from real images and were pasted randomly on a similar background, while the segmentation masks of seeds are also created. Kharin generated 3D shapes of nanoparticles from extracted textures of real EM data,^24^ with the particles positioned onto a 3D environment and backgrounds extracted from the micrographs. The obtained annotated dataset was employed to train a model for particle detection.

Likewise, Polyanichenko et al.^25^ generated synthetic data based on 3D models of metal–organic frameworks and trained a model to detect and analyze such structures in real time. Due to the extreme complexity and diversity of nanoparticle systems in terms of shape, size and spatial distribution, various textures, or occlusions, it is essential to develop a versatile, system-agnostic, and configurable program that can generate high-quality annotated datasets for DL model training.

In this paper, we first demonstrate a DR-based tool for generating synthetic images of nanoparticle systems with varying shapes along with their instance segmentation masks. The input can be as small as a single image/mask pair, from which the program extracts the background and ROI regions and applies various transformations to generate diverse and customized datasets for DL model training. The generated dataset was used for supervised learning tasks including nanoparticle classification and nanoparticle segmentation. Next, CV-based tools were developed to extract the size of the NPs and to generate particle size distribution plots. The trained models were further deployed into our cloud-based platform, Virtual Minds (ViMi) Labs,^26^ which offers an interface for high-throughput image analysis for functional energy materials.

## Methods

**Figure 2:**
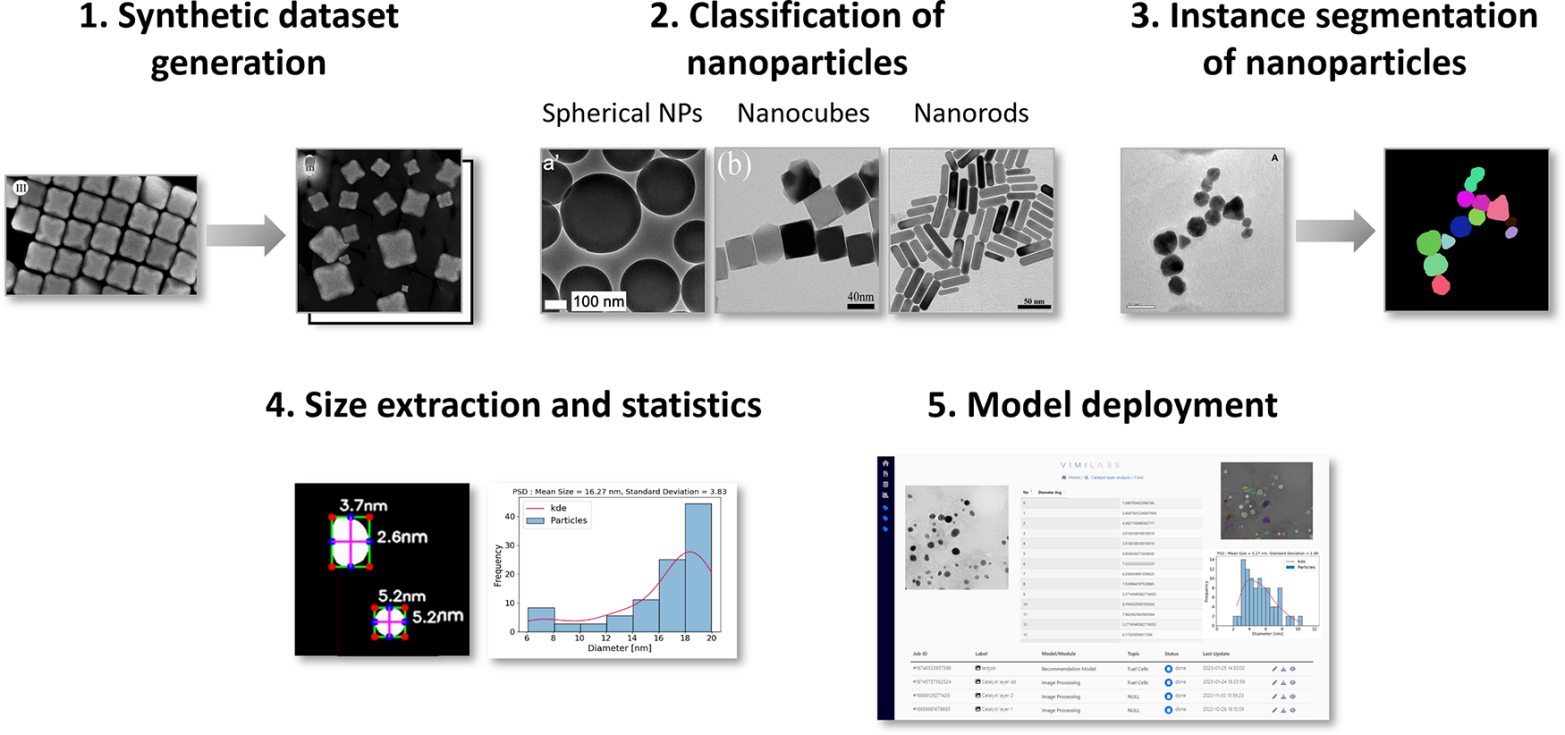
Methodical pipeline for deep learning-based analysis of nanoparticles. The pipeline consists of five main steps. (1) Synthetic dataset generation: a small number of annotated images are utilized to generate a synthetic dataset with the UTILE-Gen tool. (2) Shape classification: a classification model is trained to identify the shape of nanoparticles in images. (3) Instance segmentation model training: instance segmentation models are trained using the synthetic dataset. (4) Particle size and shape extraction and statistics: the predicted masks from the segmentation models are used to extract the size and analyze the shape of individual particles based thereon a statistical analysis is performed. (5) Web app deployment: the pipeline is deployed as a web application, offering access to users for nanoparticle analyses.^27^

Figure 2 demonstrates the general workflow for the automated nanoparticle analysis by utilizing our DL-based approach. It involves synthetic image generation to expand the annotated training datasets, supervised learning for image classification and particle segmentation, automated size measurement on predicted ROI, and statistics and visualization of results, followed by model deployment into the imaging platform.

As input to generate the synthetic images, we used the open access Electron Microscopy Particle Segmentation (EMPS) dataset.^27^ It consists of 465 micrographs and their corresponding manually annotated labels for segmentation model training. The dataset contains diverse particle shapes, sizes, textures, and distributions. To apply the methodical workflow in Figure 2 to various particle systems, we inspected the EMPS dataset based on the shape of the particles and collected the images in 3 different classes, namely, the spherical NPs, nanocubes, and nanorods.

### Synthetic Image Generator

**Figure 3:**
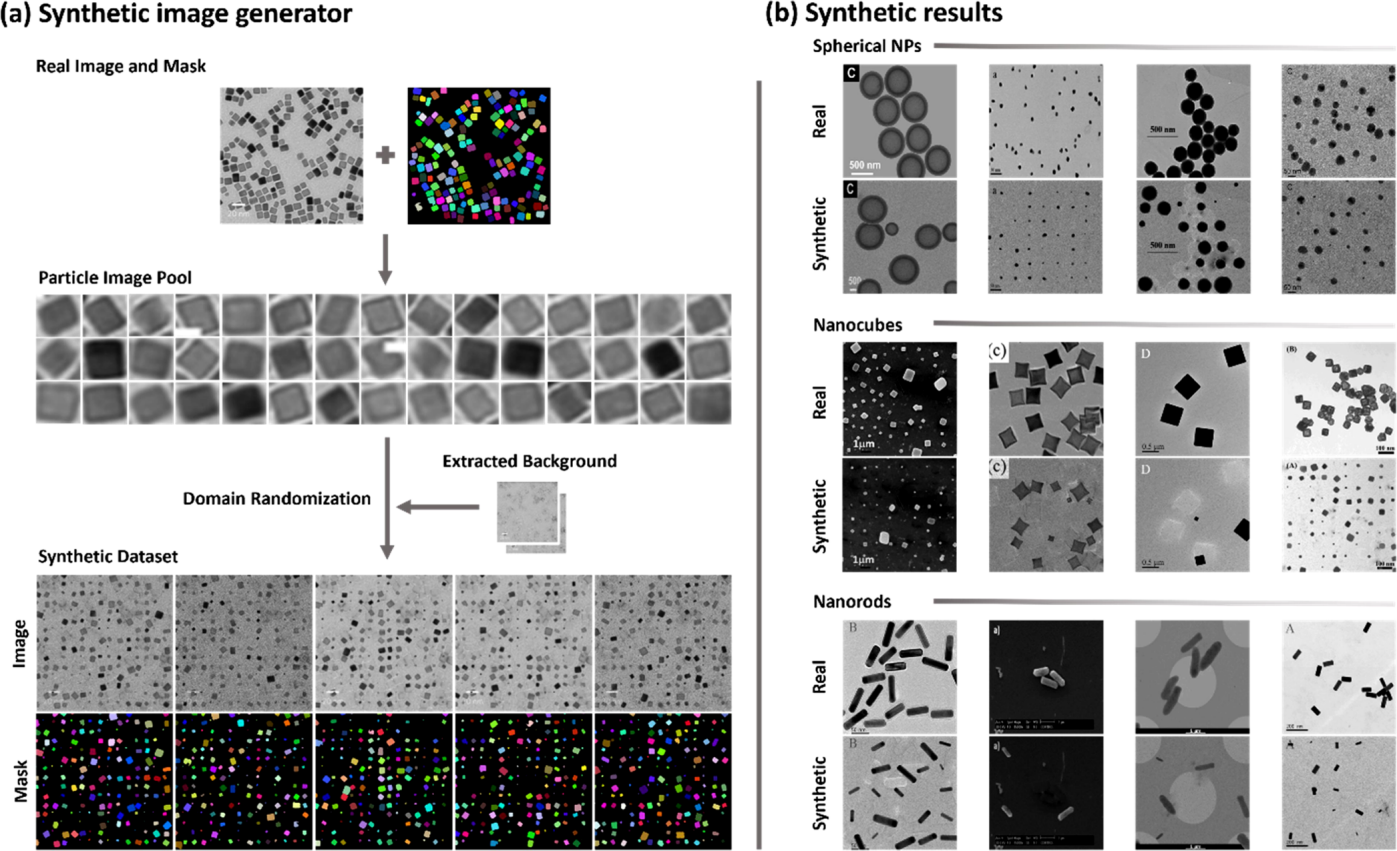
(a) Schematic pipeline for synthesizing images based on image/mask pairs. (b) Examples of real images from the EMPS dataset in three different classes of nanoparticles,^27^ along with the generated synthetic image using the tool developed in this work.

As shown in Figure 3a, our algorithm takes the EM image and its segmentation mask as the input, automatically extracts the ROIs and background, and saves them into their pools. Thereafter, the software applies customized random transformations to locate the ROIs on the background; simultaneously, it generates the corresponding masks for the synthetic image through the same transformations.

**Figure 4:**
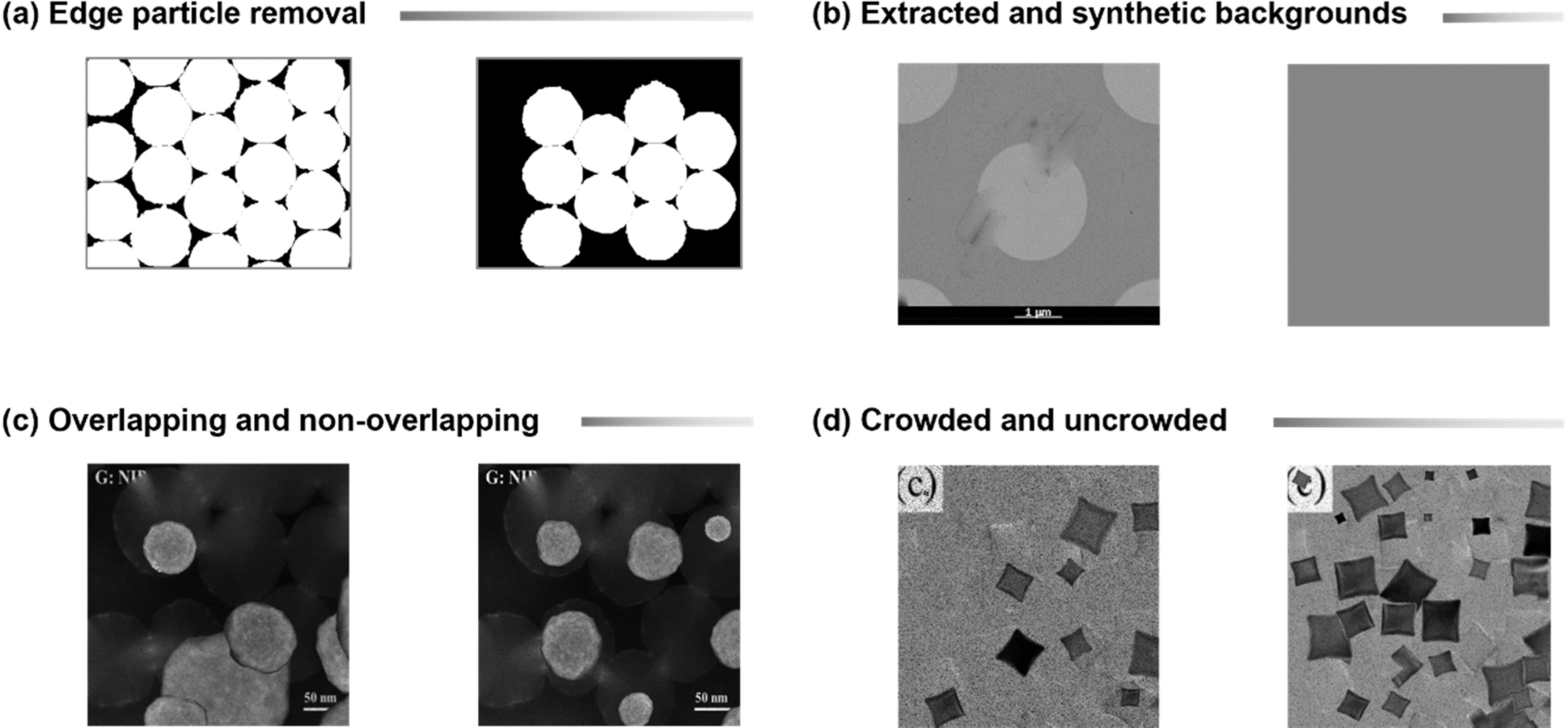
Schematic of synthetic image generator tool capabilities. (a) Edge particle removal: artifact reduction by removing edge particles. (b) Inhomogeneous background extraction and synthesis: extraction or creation of new inhomogeneous backgrounds. (c) Synthetic particle arrangements: representation of different arrangements for synthetic particles. (d) Custom crowding selection: feature for selecting the level of crowding in synthetic images.

For the first step, ImageJ API and the PymageJ library were used to locate the position of ROI from the annotated mask and to crop the ROI into individual image files for creating the particle image pool.^23^ Chopped ROI due to overlapping 2D view or when located at the boundaries of the real image affects the quality of synthetic data, and therefore, they need to be removed from the ROI pool. To address the former issue, the size of individual ROI was quantified in pixels and a tunable parameter was set in the program to eliminate all particles below a certain size. Here, the particles with less than 60% of the average size of ROIs were removed from the pool to remove the truncated overlapping particles. As shown in Figure 4a, in order to filter the chopped particles at the boundaries from the pool, the pixel values from the border were inspected and the ROI with values detected at the boundaries of the image was removed from the mask. This process could prevent the extraction of chopped boundary particles.

To generate the background canvas, as shown in Figure 4b, two different methods were implemented in the software to address the cases with homogeneous and inhomogeneous background textures. The first one employs the inPaint() function from the OpenCV library where the annotated objects in the mask can be removed from the real image and substituted by a neutral hue based on the surrounding pixels of the object. The result generates an empty canvas and keeps the features of the original image such as the background inhomogeneities, or the scale bar. The second method identifies the background from the real mask and computes the average background pixel’s intensities from the corresponding real image. The mean intensity can then be used to create a canvas for the synthetic images. The latter is more suitable for systems with homogeneous backgrounds.

Next, a series of hyperparameters were specified to customize the synthetic images including options for creating the overlapping vs nonoverlapping ROIs (Figure 4c) or specifying the number of particles in the image (Figure 4d). To generate the image/mask pairs, first, a background is randomly taken from the background pool along with a black frame for the mask. Next, a particle is randomly picked from the particle pool, rotated, and rescaled within a specified range to introduce variations. Then, the particle is pasted onto the background, and a particle filled with a unique color is placed into the mask canvas in the same location. For overlapping systems, the ROIs are randomly pasted into the canvas, while for nonoverlapping instances, the canvas is divided into a grid based on the number of particles and populated afterward to avoid overlapping. This process can be repeated until the required particle count per image is obtained. Next, salt and pepper noise and Gaussian blur filter are added for smoothing and enhancing the realism of the images. Finally, the image/mask pairs are converted to TIFF files and saved in a folder structure for deep learning model training. The output size of the generated images and masks is set by default to 1024 × 1024 pixels.

Table 1 summarizes the hyperparameters in the program to generate the customized dataset. To prepare the dataset for supervised learning tasks in this work, the number of particles per image was selected as almost the same number as in the original image to avoid over- and undercrowded images. Salt and pepper noise and Gaussian blur were set to 0.1 and 0.3, respectively, and the range scaling factor was set at 0.3 and 2 for the lower and upper limits, respectively.

**Table 1:** Description of the Input Parameters for the Creation of Customized Datasets with the Synthetic Dataset Generator Tool

hyperparameter	input	description
**dataset size**	integer	number of generated synthetic image/mask pairs
**particles per image**	integer range	random number inside the range of objects per image
**overlapping particles**	boolean	if true, the objects are pasted randomly in a grid with minimal overlap
**synthetic backgrounds**	boolean	if true, the mean background intensity is extracted, and a synthetic background is added to the pool
**salt-pepper noise**	upper-limit float	random SP-noise between 0 and the given limit is applied to every image
**gaussian blur**	upper-limit float	random GB between 0 and the given limit is applied to every image

### Supervised Model Training

The synthetic data was used to train ConvNet-based image classification and instance segmentation models. In the typical classification architectures, an image is provided as input and the probability distribution over the different predefined classes is predicted as output. The image is a 3D tensor (column × row × channel) and the ConvNet applies a set of convolutions defined via learnable kernels (filters or feature detectors) by sliding over spatial locations of the image to generate the transformed representations or activation maps. The activation maps have smaller spatial dimensions and larger depth in the channel dimension. Each generated activation map channel learns to respond to certain visual features of the image—from low- to high-level features as it goes deeper into the convolutional layers.

The convolution operations are typically followed by applying activation functions and pooling operations. Activations functions such as Rectified Linear Units (ReLU) introduce nonlinearities into the layer-wise signal transformation. After an image is passed through a series of convolutional layers and the fully connected layer, the classifier can be applied to the 1D feature vector to generate the probability distribution over each class and the one with the highest probability is mapped as the class label for the image. During the model training, the gradient of the loss function is computed for each weight in the convolutional kernels using backpropagation, and the weights are updated to minimize the loss function.

To classify the images into different nanoparticle systems, we used transfer learning and fine-tuned a pretrained model on our synthetic dataset. A pretrained model can be initially trained on a large generic natural image dataset and then be used to extract useful generic features from the image. In our work, we employed the Xception^28^ model pretrained on the ImageNet dataset.^29^ For classification, the synthetic dataset of nanoparticles was generated using 179 real images in the three different classes—spherical-, cubic-, and rod-shaped. The dataset was split into two sets for training, including 126 images from which 750 images were created synthetically, and 53 images for validation.

**Figure 5:**
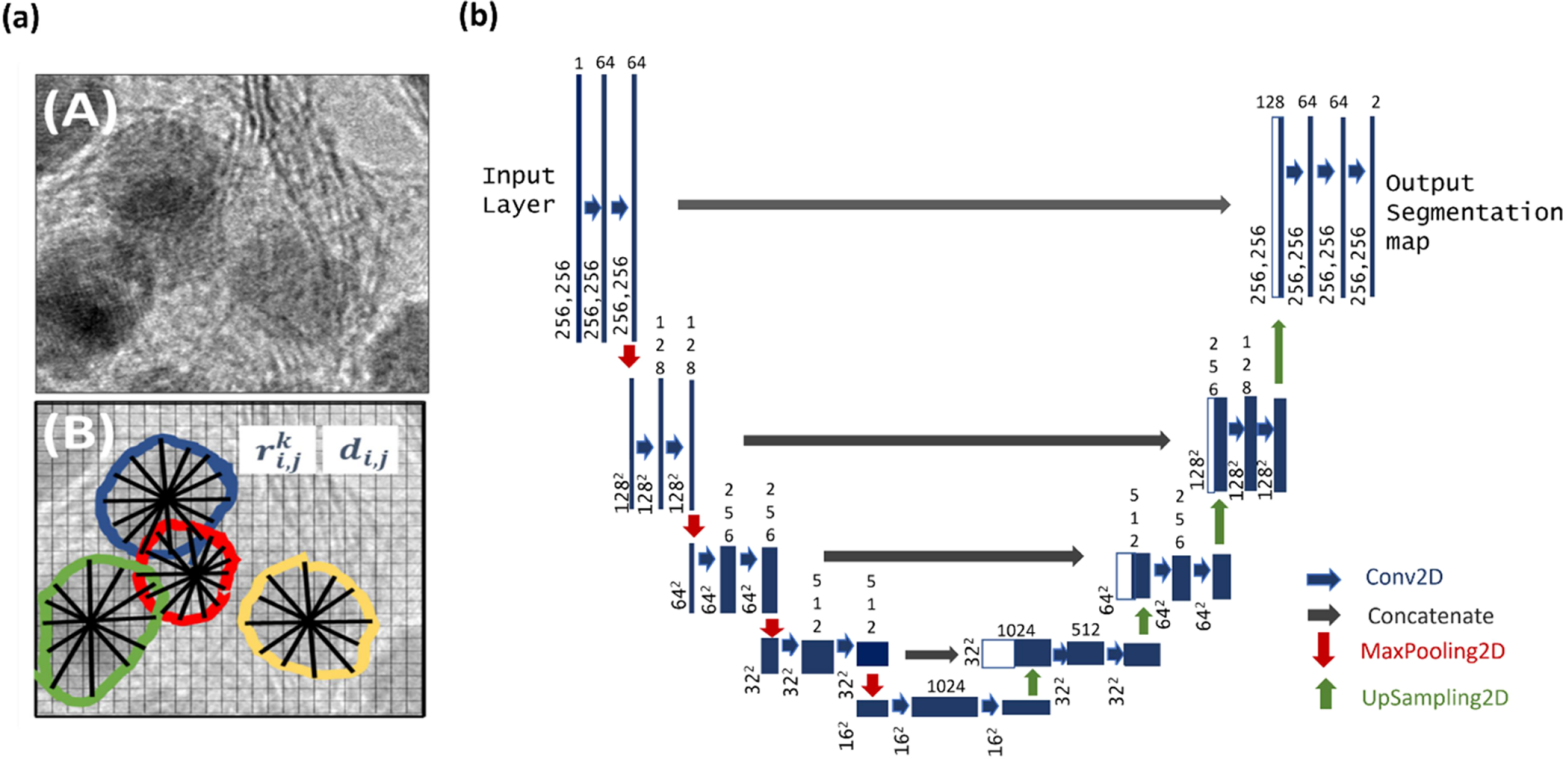
(a) Sample EM image depicting overlapping particles. The StarDist model^16^ captures star-convex polygons for each particle via radial distances (*r*_i,j_) and object probabilities (*d*_i,j_) for the pixel i,j. (b) The U-Net architecture.^15,16^

For the instance segmentation task, the U-Net architecture with StarDist loss function was chosen (Figure 5).^15−17^ Standard architectures, for instance segmentation, involve fully convolutional networks, which first perform convolution and down-sampling operations to extract the features (encoder), followed by up-sampling and transpose convolution operations (decoder) until the starting input size is reached. The U-Net model, in addition to the described data flow, concatenates the up-sampled decoder features with the corresponding ones from the encoder (Figure 5b). The concatenation of the encoder and decoder feature maps across all resolution levels enables an improved recognition of object boundaries and edges, leading to an increased performance and more accurate output segmentation maps. The StarDist add-on to the loss function of the U-Net backbone overcomes the typical issues for the dense prediction of merged bordering particles. In StarDist, each pixel from the detected ROI is parameterized by two values, radial distances, and object probabilities (Figure 5a). For the radial distance, a star-convex polygon is fitted from the pixel position to the edges of the particle and the object probability value is calculated from the shortest distance of the pixel to the edge of the particle. Non-maximum suppression (NMS) technique is then applied to eliminate the overlapping detections with lower object probabilities leading to segmentation of individual overlapping instances.

Standard augmentation techniques^30^ were employed to expand the training set through basic image manipulations such as cropping images into patches and augmenting by rotation, varying intensity, or applying Gaussian blur. The instance segmentation models were trained using the default hyperparameter in the StarDist implementation of U-Net with the number of epochs and steps per epoch set as 200 and 100, respectively. The performance of the trained model was analyzed using the Intersection over Union (IoU) threshold. The IoU measures the number of pixels common between the manual annotated masks, the so-called ground truth, and prediction masks, obtained by the model, divided by the total number of existing pixels in both masks. A true-positive (TP) represents the case if a prediction–target mask pair for nanoparticles has an IoU score that exceeds 0.5; a true-negative (TN) is that for background. On the other hand, a false-positive (FP) indicates a predicted NP mask with no associated ground truth mask, and a false-negative (FN) indicates a ground truth NP mask with no associated predicted mask. This way, the metrics of accuracy, precision, recall, and *F*1 score are defined as
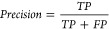

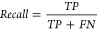

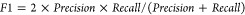
Given the segmentation maps obtained from the model, each ROI was cropped into an individual image. Next, we employed the OpenCV library to draw a bounding box around the particles and measured the pixels along with the *X* and Y directions. Real distances can then be calculated from the pixel values with image calibration. Finally, the obtained sizes for the *X* and *Y* axis were used to generate the histograms for the particle size analysis and other statistics such as perimeter, area, solidity, roundness, extent, and aspect ratios.

## Results and Discussion

**Figure 6:**
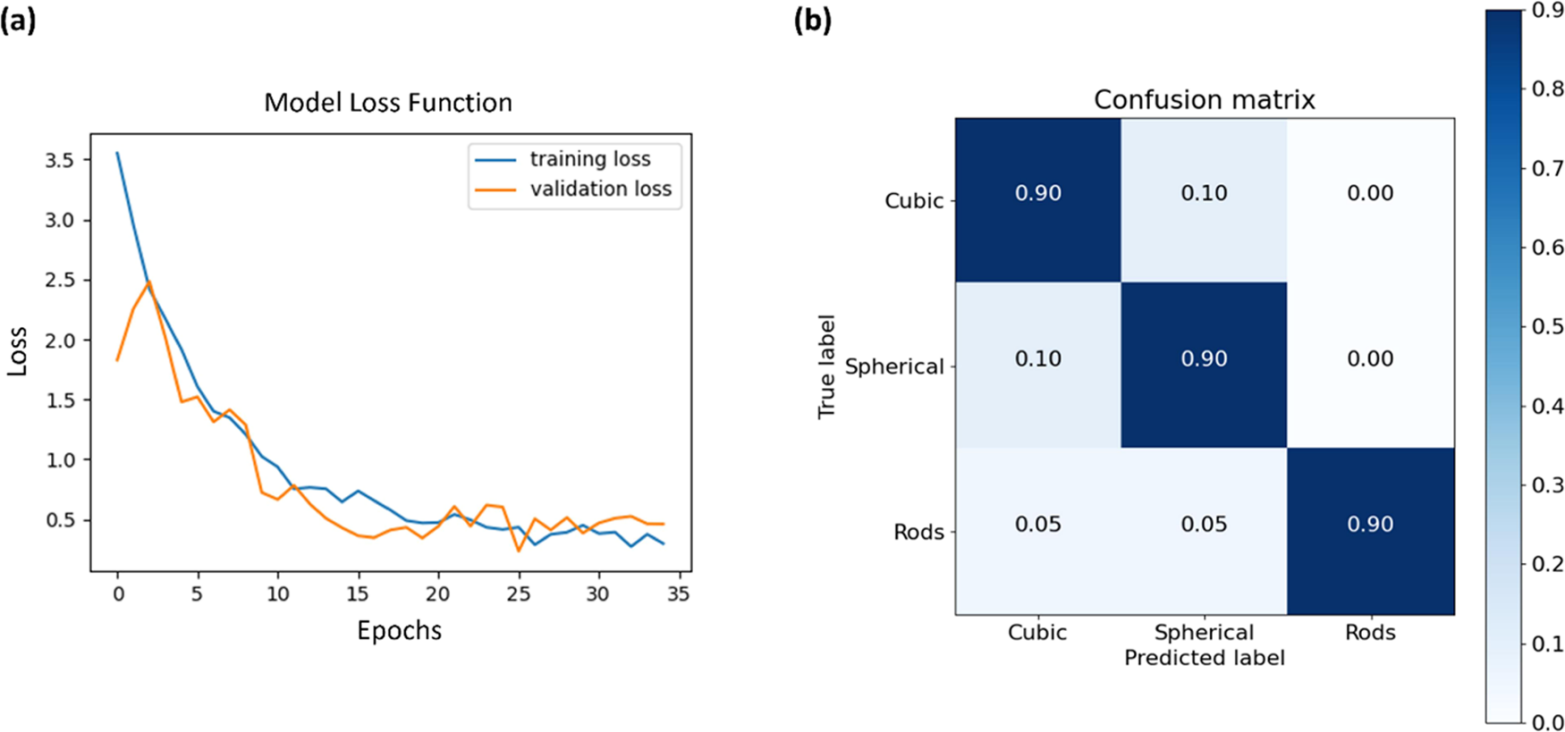
(a) Learning curves for fine-tuning the pretrained Xception model for the classification of nanoparticles into three classes: cubic-, spherical-, and rod-shaped particles. (b) Normalized confusion matrix on the test set.

For classifying nanoparticles, the comprehensive process of fine-tuning and assessing the pretrained model after dividing the data involves selecting the network architecture, initializing the weights from a pretrained model to improve the performance, and selecting the optimal learning rate to determine how large are the steps toward the optimal minimum and regularization strength. These process steps are required to minimize the loss function, which computes how large the error between the manual annotated mask and the predicted mask by the model is. Afterward, we evaluate the model using the validation set. Figure 6a shows the learning curves (validation loss vs epoch number) of the pretrained Xception model fine-tuned on our dataset. The training and validation loss curves displayed no signs of overfitting/underfitting during the model training, On the validation set, the model achieved an accuracy of 91%. Figure 6b shows the normalized confusion matrix for the classifier on the test set, which was not used as input for the synthetic dataset generator. The x-axis shows the predicted labels, and the y-axis shows the true labels. The classification accuracy of ∼90% can also be seen in the confusion matrix.

In this study, we compared state-of-the-art models and augmentation techniques by assessing the impact of synthetic data on the prediction performance of models in cases with limited data. The Xception model and the ConvNeXtBase model,^31^ a state-of-the-art deep learning architecture, were trained on two datasets: one consisting of 15 real images per class and mixed dataset consisting of 15 real images per class and 5 synthetic images per real image. Furthermore, we evaluated the use of advanced augmentation techniques, such as RandAugment and MixUp,^32,33^ to determine their effects on model performance in comparison to the base models.

Table 2 compares the classification accuracies from the models trained on real images, as well as mixed real and synthetic images using various augmentation techniques. The Xception model demonstrated improved accuracy from 30 to 64% when advanced augmentation methods were applied on real images,^32,33^ while the best performance was achieved by combining synthetic and real images from 68 to 75%. On the other hand, the classification accuracy of more advanced architectures like ConvNeXtBase network showed a slight decrease in performance when subjected to the use of synthetic data, saturating at 85%, while the employment of state-of-the-art augmentations could slightly increase the performance to 89% on our dataset.

**Table 2:** Comparison of Model Accuracy Using Real Data, Mixed Real and Synthetic Data, and Various Augmentation Techniques for the Xception and ConvNeXtBase Networks

	model accuracy [%]	
model	standard augmentation real/Mix	MixUp + RandAug Real/Mix
Xception	30/68	64/75
ConvNeXtBase	87/87	89/85

**Figure 7:**
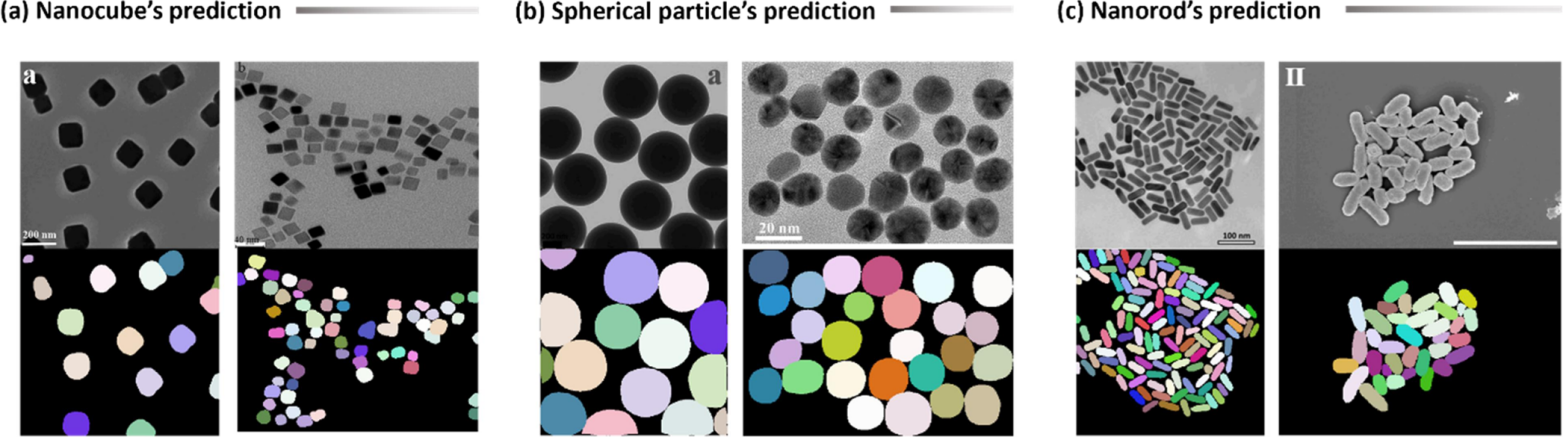
Prediction of segmentation model on test images for various nanoparticle classes: (a) nanocubes, (b) spherical nanoparticles, and (c) nanorods.^27^

For the instance segmentation task, the images in each class were split into approximately 80% for the training set and 20% for the validation set. Next, using the synthetic image generator tool, the training set was expanded by 500%. Afterward, the U-Net model was trained on the real images as well as the expanded training set. Table 3 summarizes the performance of the model for the two cases by calculating the metrics for precision, recall, and *F*1 score. For all particle categories, up to 13% improvements in the performance metrics were obtained using synthetic data. Figure 7 demonstrates a few examples of the segmentation results on the test set, for the model trained on the expanded dataset, and for the three different particle categories.

**Table 3:** Performance Comparison of Segmentation Model Trained on Only Real Data versus the Mixture of Real and Synthetic Data

dataset	precision [%] real/mix	recall [%] real/mix	*F*1 [%] real/mix
nanocubes	89/90	72/78	79/84
nanoparticles	81/94	85/92	84/93
nanorods	86/92	87/93	87/93

To further analyze the utility of the synthetic image generator tool, further experimentations were conducted by starting from the worst-case scenario where only one annotated image is available. Even though in this extreme case, training a DL model is not relevant, by generating synthetic data, we can enable automation in the image analysis. To test this, one image was randomly selected starting from three different cases of black NPs, gray NPs, and ordered nanorods, and a synthetic dataset of each image was generated consisting of 50 synthetic image/mask pairs. Afterward, for each class, a model was trained on synthetic data. The remaining images in each class were used for validation of the results. As summarized in Table 4, acceptable performances were obtained for the models only trained on the synthetic data in a most difficult scenario where only one annotated real image/mask pair is available. This utility of the tool means that with just a minimal manual annotation effort, we can obtain an acceptable performance of the DL model for the segmentation of various nanoparticle systems.

**Table 4:** Instance Segmentation Performance Metrics of Three Use-Cases for Models Trained on 50 Synthetic Images Generated from One Real Image

dataset	precision [%]	recall [%]	*F*1 [%]	validation set size
gray NPs	88	63	73	39
black NPs	91	93	92	62
ordered nanorods	80	75	77	8

We further evaluated the performance improvements by varying the number of real image/mask pairs as input for the synthetic image generator. For this test, the largest class dataset was employed, which consists of 93 images of spherical NPs, which were split into 30 images for training and 63 images for validation. From the 30 real image/mask pairs for training, four datasets consisting of 5, 10, 20, and 30 images were created on which the synthetic image generator tool was applied to expand the training set by 500%. Table 5 shows the *F*1 score obtained from this experimentation. Consistently, by increasing the number of real images, the *F*1 score increases from 36% for 5 images as the training set to 88% for 30 real images. Expanding the training set by generating 5 synthetic images per real image, the *F*1 score is significantly improved from 36% for 5 real images to 74% on the corresponding expanded training set. Likewise, the *F*1 score was improved from 63, 77, and 88% when the model is trained on 10, 20, and 30 real image/mask pairs to 86, 88, and 92% on the expanded dataset, respectively.

**Table 5:** *F*1 Score Comparison of Segmentation Models Trained on Different Numbers of Input Real Image/Mask and of the Expanded Synthetic Dataset

*F*1- score [%]	5 real images	10 real images	20 real images	30 real images
0 synth images per real image	36	63	77	88
5 synth images per real image	74	86	88	92

Following the overall workflow to automate the analysis as shown in Figure 2, particle sizes, areas, aspect ratios, solidities, orientations, extents, perimeters, and roundness can be extracted in step 4 with a separately developed CV tool.

The area of the region of interest (ROI) is quantified by the count of nonzero pixels, representing the occupied space by the ROI. The equivalent diameter is calculated based on the area, representing the diameter of a circle with the same area as the ROI.

The aspect ratio of the ROI is determined as the ratio of the width to the height of the individual object of interest, providing an understanding of the shape of the ROI. Solidity measures the compactness of the ROI, determined by the ratio of the ROI’s contour area to the convex hull area. The convex hull refers to the smallest convex polygon that can completely enclose the contour. Orientation describes the angle at which the ROI is tilted. It is determined by fitting an ellipse to the contour and reporting the angle of this fitted ellipse. Extent quantifies the ratio of the contour area of the ROI to its bounding rectangle area, indicating how much of the bounding rectangle the ROI occupies. The perimeter is calculated as the arc length of the contour of the ROI, signifying the length of its boundary. Roundness, or circularity, is a measure of how closely the shape of the ROI resembles a perfect circle. It is computed as the ratio of 4π times the area to the square of the perimeter.

**Figure 8:**
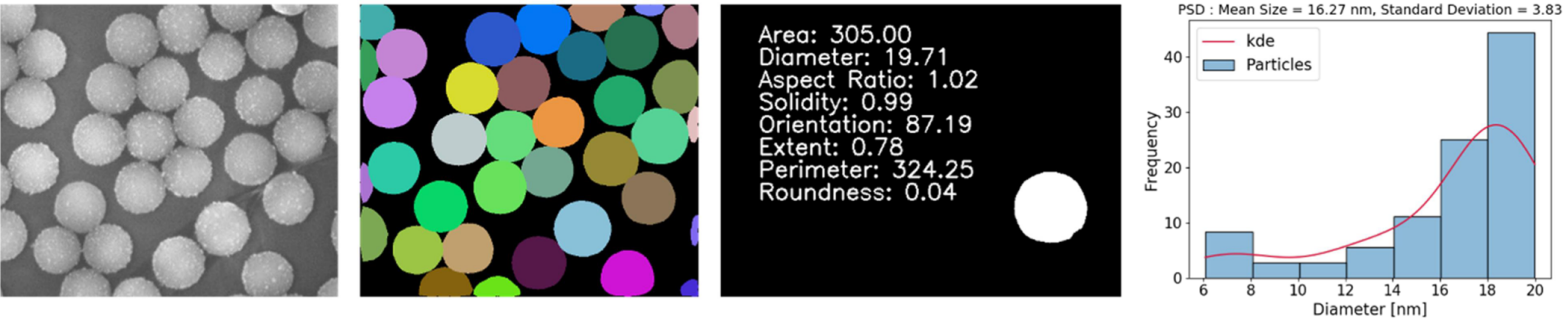
Automating particle size distribution analysis using the deep learning-based workflow based on instance segmentation and measurements of nanoparticles in EM micrographs.^27^

From this result, the particle size histograms can be generated using typical visualization tools with appropriate binning size and fitting function, as exemplified in Figure 8.

**Figure 9:**
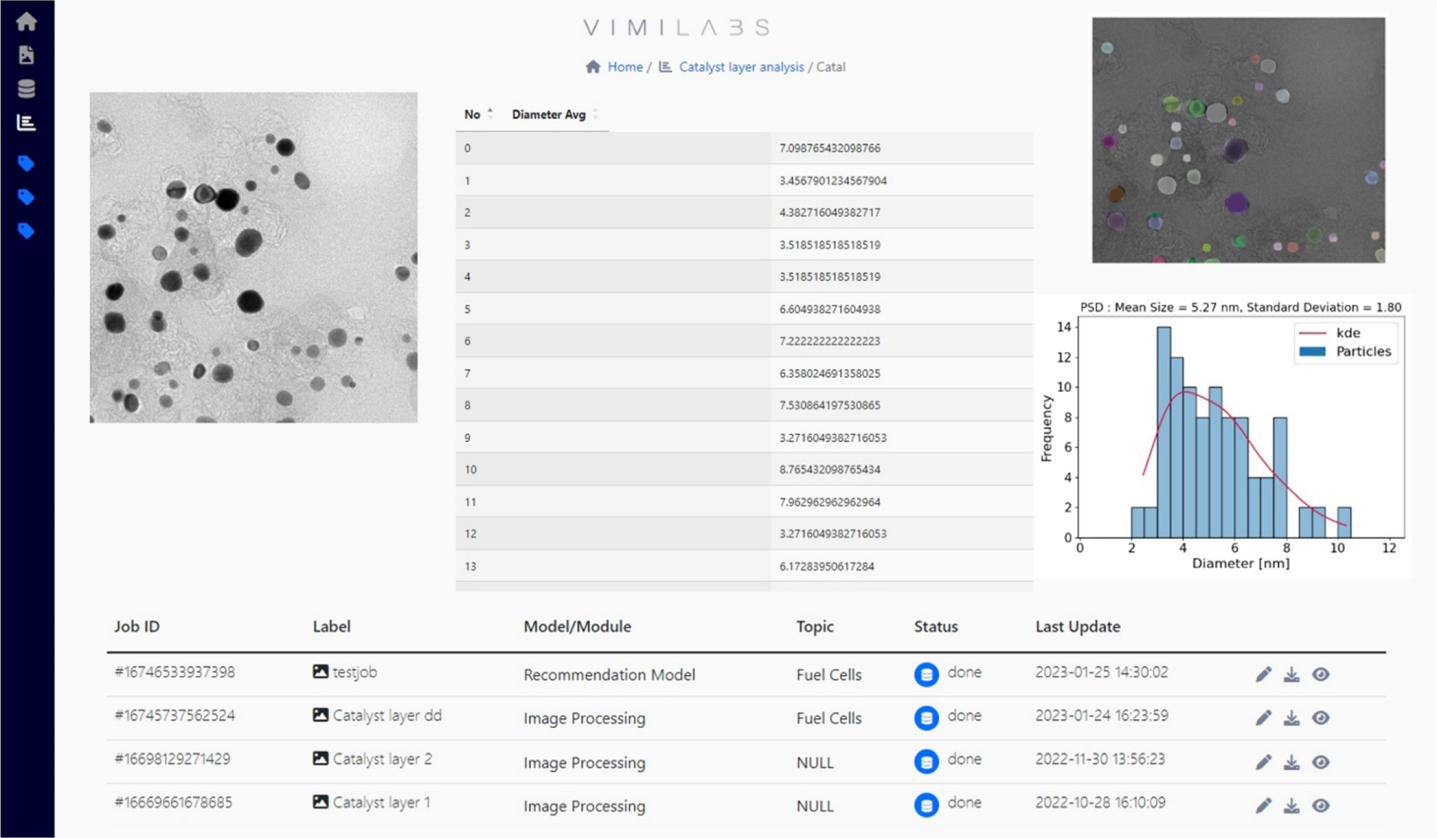
Example of the utilization of the trained models for particle size distribution analysis deployed in the ViMi labs platform.^26^

The developed DL-based workflow described in this paper was partly deployed into the Virtual Minds Labs (ViMi Labs, vimi.ai). As shown in Figure 9, the imaging module of ViMi labs currently allows the users to upload bulk EM images of electrocatalyst materials and obtain the particle size distribution (PSD) and other distributions plots in a noticeably short amount of time (∼10 s processing for each image), while the manual or semiautomated PSD analysis can take up to several hours depending on the number of particles per image. To fit the obtained histogram, the kernel density estimation method and log-normal distribution were tested, giving the kernel density estimation method a superior fit. However, the user of the code will have the option to choose among fitting methods and obtain the best estimate of the underlying probability distribution. The platform provides a visual representation of the results, including the uploaded image, individual measurements of the objects of interest, particle analysis, and mask of the model for prediction verification. Results can also be downloaded as a .csv file.

## Conclusions

This work presented a multistep DL-based methodical workflow to automate microscopy image analysis in nanoscience. It involves generating synthetic annotated imaging data for nanoparticles with varying shapes using domain randomization. Afterward, we trained DL models on the augmented data for classification and instance segmentation, followed by computer vision-based automated measurement of particles, and finally deployment of the model into our cloud-based web application (vimi.ai).

Our synthetic image generator tool is customizable and nanoparticle shape agnostic, which outputs image/mask pairs for image segmentation model training tasks. The software can create a large amount of data employing domain randomization and provides several hyperparameters such as noise, blurring, scaling factor, and number or distribution of the particles which enables the application for various use-cases of interest. The data augmentation enabled by the synthetic image generator tool was extensively evaluated by comparing the performances of various trained deep learning models. Accurate particle classification and segmentation can pave the way for the accelerated characterization of nanomaterials. Future work should focus on developing real-time image analysis tools and deployment into web, mobile, or desktop applications.

## Data Availability

The software for the synthetic image generator tool is available at https://github.com/andyco98/UTILE-Gen.
